# Fatigue Failure Mechanism and Crack Growth Behavior of Ti-6Al-4V ELI Titanium Alloy Welded Joints

**DOI:** 10.3390/ma19112301

**Published:** 2026-05-29

**Authors:** Jiajun Liu, Yu Li, Shao-Shi Rui, Wei Chen, Chengqi Sun

**Affiliations:** 1 School of Engineering Science, University of Chinese Academy of Sciences, Beijing 100049, China; liujiajun@imech.ac.cn; 2 State Key Laboratory of Nonlinear Mechanics, Institute of Mechanics, Chinese Academy of Sciences, Beijing 100190, China; ruishaoshi@imech.ac.cn; 3 China Ship Scientific Research Center, Wuxi 214082, China; 19109672721@163.com (Y.L.);

**Keywords:** Ti-6Al-4V ELI titanium alloy, welded joints, fatigue failure mechanism, crack growth behavior, mixed-mode I–II, equivalent stress intensity factor range

## Abstract

**Highlights:**

**What are the main findings?**

- Welded joints show lower axial fatigue strength than the base material.
- Fatigue cracks mainly initiate from welding-induced gas pores.
- Welded joints exhibit higher near-threshold crack growth resistance under mode I loading.
- A modified equivalent stress intensity factor range model correlates mode I and mixed-mode I–II crack growth data.

**What are the implications of the main findings?**

- Gas pores are key defects controlling fatigue crack initiation in welded joints.
- Fatigue degradation is mainly related to crack initiation from welding defects.
- Near-threshed crack growth rate in welded joints is slower than that in base material.
- New models are needed for correlating the crack growth data of welded joints under mode I and mixed-mode I–II loadings.

**Abstract:**

Titanium alloy welded joints are key parts of deep-sea pressure hulls, which are subjected to fatigue loadings in service. In this study, axial fatigue tests, mode I fatigue crack growth tests, and mixed-mode I–II fatigue crack growth tests were conducted on the Ti-6Al-4V ELI titanium alloy welded joint, and its fatigue failure mechanism and crack growth behavior is investigated and compared with the base material. The results show that the *S–N* curve of Ti-6Al-4V ELI titanium alloy welded joints has a very similar slope as the base material, but its fatigue performance is lower than the base material. However, the welded joints exhibit a higher resistance in the near-threshold region under mode I loading compared to the base material. Scanning electron microscope observation indicates that the fatigue crack mainly initiates from gas pores during welding for the Ti-6Al-4V ELI titanium alloy welded joints. Under mixed-mode I–II loading, the stress intensity factor range component $\Delta K_{I}$ of welded joints is higher than that of the base material, and an equivalent stress intensity factor range model is proposed to describe the crack growth rate under both mode I and mixed-mode I–II loadings. The new model incorporates a parameter dependent on the mode mixity ratio defined by $\Delta K_{II}/\Delta K_{I}$ in this paper, and it unifies the crack growth data well under mode I and mixed-mode I–II loadings. The paper indicates that the gas pores during welding are an important factor for the poor fatigue performance of Ti-6Al-4V ELI titanium alloy welded joints.

## 1. Introduction

With the increasing demand for deep-sea resource exploration and exploitation, the development of deep-sea manned equipment has attracted considerable attention. As the core load-bearing component of deep-sea vehicles, the pressure hull is subjected to extremely high hydrostatic pressure during service, and experiences repeated load fluctuations during the diving–cruising–surfacing cycle, making fatigue performance a key concern for structural safety. Owing to its low density, high specific strength, excellent corrosion resistance in seawater, and good fracture toughness, the Ti-6Al-4V ELI titanium alloy has become an important material for deep-sea pressure hull structures [1,2].

In the fabrication of large and complex pressure hull structures, welding is unavoidable. However, the welding thermal cycle can significantly alter the microstructure of the fusion zone (FZ) and heat-affected zone (HAZ), and also introduce welding defects such as gas pores and lack of fusion. In addition, welding-induced residual stress may affect the fatigue behavior of welded components. These factors can influence fatigue crack initiation and propagation, and affect the structural integrity and service reliability of welded components [3]. Therefore, understanding the fatigue behavior of Ti-6Al-4V ELI titanium alloy welded joints is of great engineering importance.

Existing studies have examined the fatigue behavior of titanium alloy welded joints from different perspectives. For axial fatigue, Rajan et al. [4] reported that the fatigue performance of linear friction-welded Ti-6Al-4V titanium alloy joints was slightly inferior to that of the base material (BM), with fracture occurring in the welded region. Long et al. [5] compared two kinds of high-energy-beam welded TC4 joints and showed that the fatigue performance depended strongly on the welding process. In terms of fatigue crack growth, Tsay et al. [6] found enhanced crack growth resistance in the FZ and HAZ of Ti-6Al-4V titanium alloy laser welds, whereas Long et al. [7] investigated the initiation and propagation mechanisms in ultra-thick titanium alloy vacuum electron beam welded joints. These results indicate that the fatigue performance of titanium alloy welded joints is highly sensitive to weld-region microstructure, defect characteristics, and loading conditions [8].

In actual service, deep-sea pressure hulls are subjected to multi-axial fatigue loading. This will result in mixed-mode crack growth. Mixed-mode fatigue crack propagation has been studied in titanium alloys and welded structures using compact tension–shear specimens and related loading fixtures [9,10,11]. These studies have shown that crack path and crack growth rate are strongly affected by the mixity mode ratio, and that equivalent stress intensity factor range approaches can often be used to correlate mixed-mode crack growth data. Nevertheless, most existing studies have focused on homogeneous materials or specific welding regions, while systematic comparisons between welded joints and BM covering pure mode I crack growth and mixed-mode I–II crack growth remain limited.

In this study, axial fatigue tests, mode I fatigue crack growth tests, and mixed-mode I–II fatigue crack growth tests were first conducted for Ti-6Al-4V ELI titanium alloy welded joints and compared with the BM. Then, fractographic observations were used to identify crack initiation characteristics for Ti-6Al-4V ELI titanium alloy welded joints. Finally, the equivalent stress intensity factor range was investigated for crack growth in Ti-6Al-4V ELI titanium alloy and its welded joints under mode I and mixed-mode I–II loadings.

## 2. Materials and Methods

### 2.1. Test Material

**Figure 1:**
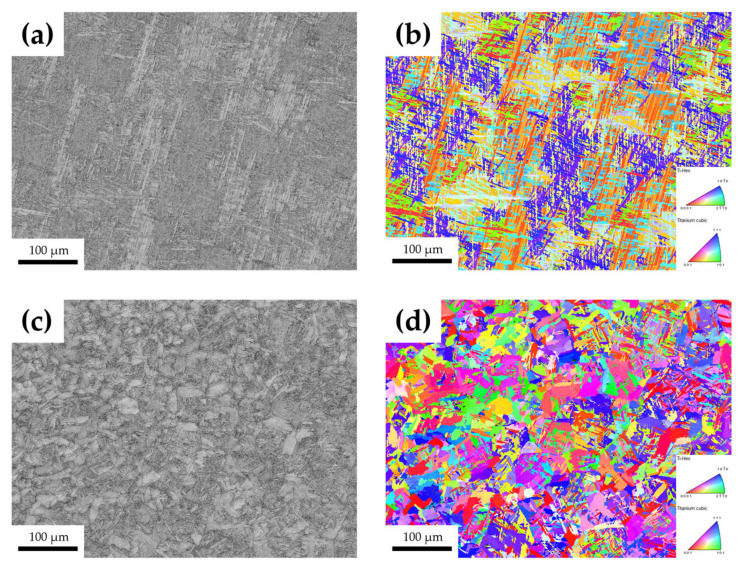
Microstructure of the Ti-6Al-4V ELI titanium alloy FZ and HAZ: (**a**) Band contrast (BC) map of the FZ; (**b**) Inverse pole figure (IPF) of the FZ; (**c**) BC map of the HAZ; (**d**) IPF of the HAZ.

The material used in this study was Ti-6Al-4V ELI titanium alloy, and its chemical composition is listed in Table 1. The microstructure and basic mechanical properties of the BM have been reported previously [12]. The welded joint was fabricated by a layer-by-layer deposition welding process. Figure 1 shows the microstructure of the FZ and the HAZ by electron backscatter diffraction (EBSD). The FZ is composed of coarse prior-β grains, and the interiors of these grains are filled with acicular α′ martensite. The HAZ is located between the FZ and the BM, and its microstructure exhibits a gradual transition from acicular α′ martensite to the equiaxed α + β duplex structure. The region adjacent to the fusion line contains coarse acicular α′ martensite, whereas the region closer to the BM consists of equiaxed α phase and transformed β.

### 2.2. Specimen Design and Preparation

**Figure 2:**
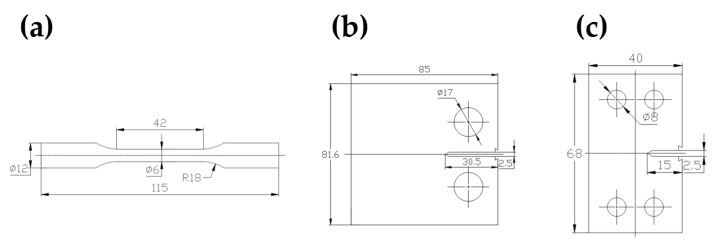
Geometries of the three types of fatigue specimens: (**a**) Round-bar specimen; (**b**) CT specimen; (**c**) CTS specimen.

Three types of fatigue specimens were used in this study. Standard round-bar specimens were employed for axial fatigue tests, standard compact tension (CT) specimens were used for pure mode I fatigue crack growth tests, and compact tension–shear (CTS) specimens were used for mixed-mode I–II fatigue crack growth tests. The round-bar specimens were designed with reference to ASTM E466 for axial fatigue testing, and the CT specimens were designed with reference to ASTM E647 for fatigue crack growth testing. The CTS specimens were designed according to the modified Arcan fixture used for mixed-mode I–II loading. The geometries of the three types of specimens are shown in Figure 2.

**Figure 3:**
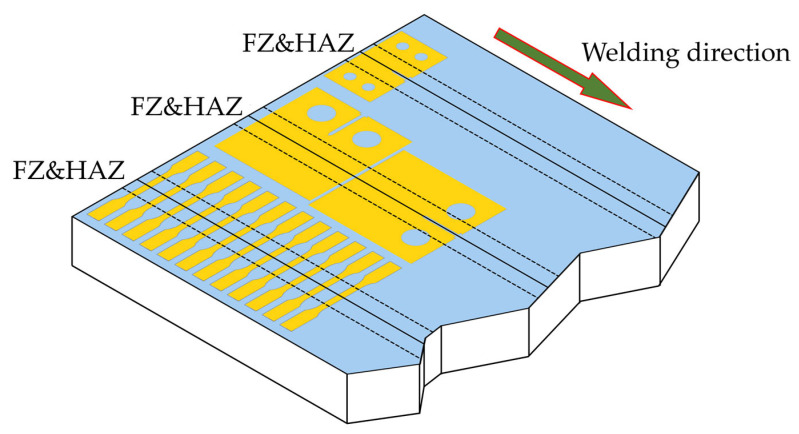
Sampling positions of the welded-joint specimens.

The sampling positions of the welded-joint specimens are shown in Figure 3. A machining allowance of 5 mm was reserved between neighboring specimens before machining. All specimens were machined from the welded joint by wire electrical discharge machining. The width of the FZ was approximately 20 mm, and the width of the HAZ was approximately 5 mm. For the CT specimens, two orientations were prepared, with the crack plane either parallel or perpendicular to the welding direction; in both cases, the FZ was located at the center of the specimen. For the CTS specimens, the FZ was parallel to the crack plane and located at the center of the specimen. For comparison, base-material crack growth specimens were also prepared, with the crack direction parallel to the rolling direction. The surfaces of the round-bar and CTS specimens were mechanically polished to a final roughness of Ra ≤ 0.4 μm.

### 2.3. Fatigue Test Methods

Axial fatigue tests were conducted in laboratory air at room temperature using a Shimadzu EHF-EV101K2-020-0A (Kyoto, Japan) fatigue testing machine. Sinusoidal loading was applied with a stress ratio of 0.05 and a loading frequency of 25 Hz. For both the BM and welded joints, six stress levels were tested, and two specimens were tested at each stress level.

Pure mode I fatigue crack growth tests were performed in laboratory air at room temperature using an MTS 319.25 fatigue testing machine. Constant-amplitude sinusoidal loading was applied with a frequency of 10 Hz and a stress ratio of 0.05. The lower frequency compared with the axial fatigue tests was to ensure stable crack-length monitoring. Before the formal crack growth test, a pre-crack with a length of 4 mm was introduced. During the test, crack length was monitored using the compliance method. For the BM, two CT specimens were tested. For the welded joints, one CT specimen was tested for each sampling orientation, namely with the crack plane parallel and perpendicular to the welding direction.

**Figure 4:**
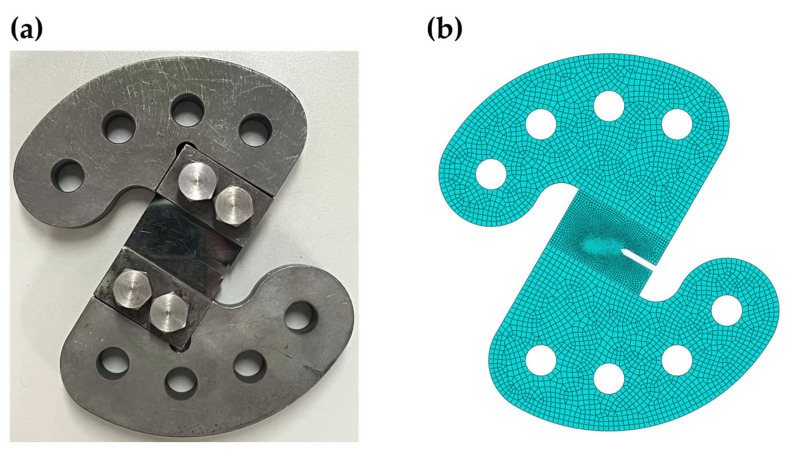
Experimental setup and finite element model for mixed-mode I–II fatigue crack growth tests: (**a**) Photograph of the modified Arcan fixture with an assembled CTS specimen; (**b**) Finite element mesh of the CTS specimen and Arcan fixture.

Mixed-mode I–II fatigue crack growth tests were carried out using a modified Arcan fixture. A photograph of the fixture and the assembled CTS specimen is shown in Figure 4a. By selecting loading holes located at different angular positions, the Arcan fixture can apply to the combined tensile–shear loading by using a conventional uniaxial testing machine [13]. The present tests followed the modified fixture design in literature [14], and the fixture was manufactured from the high-strength steel. A single loading angle of 30° was used, i.e., the loading direction was inclined by 30° to the normal of the crack plane, in order to generate mixed-mode I–II loading. Constant-amplitude sinusoidal loading was applied with a frequency of 5 Hz and a stress ratio of 0.1. The frequency and stress ratio were adopted to maintain stable loading with the modified Arcan fixture and to facilitate the crack-path image acquisition. One CTS specimen was tested for the BM, and one CTS specimen was tested for the welded joints. A pre-crack was introduced before the formal test. During crack growth, the process was recorded using a high-resolution time-lapse imaging system, and the crack length was measured after the test using image-processing software.

### 2.4. Microscopic Fractography and Stress Analysis

After fatigue tests, the fracture surfaces of welded-joint specimens were examined using scanning electron microscope (SEM). The observations focused on the overall fracture surface morphology and crack initiation region.

To calculate the stress intensity factors at crack tip during mixed-mode crack growth, a two-dimensional finite element model of the CTS specimen and the Arcan fixture was established in ABAQUS^®^, as shown in Figure 4b. Because the specimen thickness was 4 mm, which was much smaller than its in-plane dimensions, the specimen was assumed to be in a plane-stress state, and a two-dimensional model was adopted. The CTS specimen and the Arcan fixture were both modeled as linear elastic materials. The CTS specimen was assigned the mechanical properties of the Ti-6Al-4V ELI BM, whereas the Arcan fixture was modeled as a high-strength steel. The mechanical properties used in the finite element analysis are summarized in Table 2.

To reproduce the 30° tensile–shear loading condition, the specimen–fixture assembly was rotated so that the notch direction of the specimen formed an angle of 30° with the horizontal direction. The specimen and fixture were connected using tie constraints. The lower loading hole was constrained in both horizontal and vertical translation while retaining rotational freedom, and a vertically upward concentrated load was applied to the upper loading hole.

For meshing, the minimum element size in the fixture region was set to be 2 mm. The crack-tip region was locally refined to a minimum element size of 0.02 mm in order to accurately capture the stress singularity, while transitional meshing was used in the remaining regions. Mesh independence checks confirmed that this mesh size provided sufficient accuracy for the present analysis. By requesting stress intensity factor output in the ABAQUS history output and using the built-in interaction integral method, the stress intensity factor components $K_{I}$ and $K_{II}$ were obtained for different crack lengths [15].

## 3. Results and Discussion

### 3.1. Fatigue Performance

**Figure 5:**
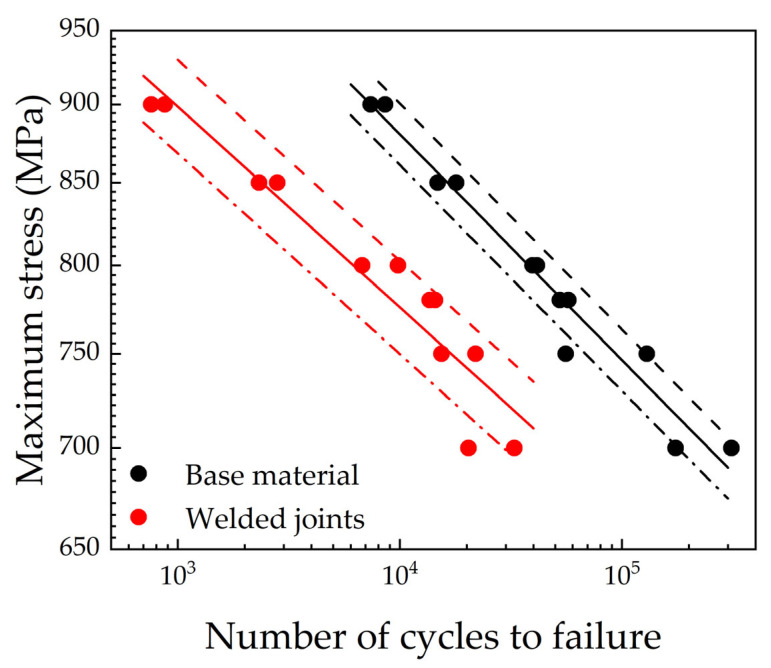
*S–N* data and predicted *P–S–N* curves of the welded joints and the BM, in which the solid curve represents the fitted *S–N* curve at 50% survival probability, while the dashed and dash-dotted curves represent the predicted *S–N* curves at 10% and 90% survival probabilities, respectively.

Figure 5 compares the *S–N* data and predicted *P–S–N* curves of the welded joints and the BM. Power-law fitting was first performed on the *S–N* data in the log–log scale using the least-squares method, and the fitted relationships are expressed as:

(1) $$S=1396*N^{-0.0638}$$

(2) $$S=1705*N^{-0.0718}$$

where *S* is the maximum stress and *N* is the fatigue life. Equation (1) corresponds to the welded joints, and Equation (2) corresponds to the BM. The coefficients of determination calculated in the log–log fitting scale are 0.911 for the welded joints and 0.961 for the BM, indicating that the power-law relationship can reasonably describe the fatigue data obtained in the present study.

Based on the fitted *S–N* relationships, *P–S–N* curves were further obtained using the method in Ref. [16]. In this paper, the logarithmic fatigue strength was assumed to be normally distributed at a given fatigue life, and *P–S–N* curves at 10%, 50%, and 90% survival probabilities are obtained as shown in Figure 5. The *P–S–N* curves of welded joints are consistently below those of the BM at the same survival probability. At *N* = 10^4^ cycles, the fitted maximum stresses at 50% survival probability are 881 MPa for the BM and 776 MPa for the welded joints, corresponding to a fatigue-strength reduction of about 11.9%. The corresponding reductions at 10% and 90% survival probabilities are 10.9% and 12.9%, respectively. The standard deviations were 0.0116 for the welded joints and 0.00763 for the BM.

### 3.2. Fractographic Analysis

**Figure 6:**
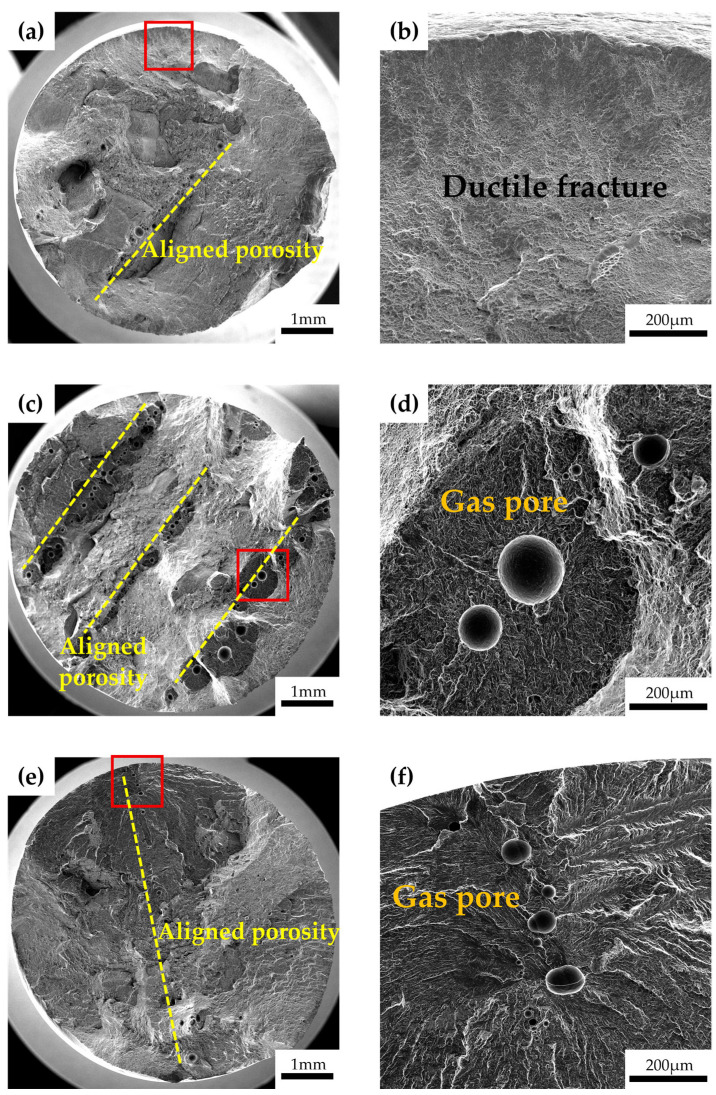
Typical SEM fractographs of failed welded joints: (**a**,**b**) *σ*_max_ = 900 MPa, *N_f_* = 760 cycles; (**a**) Overall view; (**b**) Magnified view of the boxed region in (**a**); (**c**,**d**) *σ*_max_ = 800 MPa, *N_f_* = 6758 cycles; (**c**) Overall view; (**d**) Magnified view of the boxed region in (**c**); (**e**,**f**) *σ*_max_ = 700 MPa, *N_f_* = 20,378 cycles; (**e**) Overall view, (**f**) Magnified view of the boxed region in (**e**).

Figure 6 shows typical SEM fractographs of some welded-joint specimens tested at different stress levels. The typical fracture surface characteristics of BM specimens under similar conditions have been reported elsewhere [17]. It is noted that the gauge section of the welded-joint specimens contained the BM, HAZ, and FZ. During the tests, all welded-joint specimens failed in the FZ rather than in the HAZ or BM, which indicates that the FZ is the weakest region among the BM, HAZ, and FZ under the present fatigue loading conditions.

A common feature of fracture surfaces is the presence of aligned porosity at all stress levels, in which gas pores are arranged along approximately parallel lines. This morphology is consistent with the periodic weld-layer interfaces generated during the layer-by-layer deposition welding process, and can therefore be regarded as an important defect characteristic of the welded joints.

The fracture surface morphology also changes with stress level. At high stress, the fracture surface is almost entirely covered by dimples, and no crack initiation or propagation region can be identified, indicating ductile fracture, as shown in Figure 6a,b. At intermediate stress, the fracture surface exhibits multi-site crack initiation, with several crack origins distributed across the fracture surface and fatigue propagation marks radiating from these origins; these crack origins are located mainly at gas pores, as shown in Figure 6c,d. At low stress, the fatigue crack initiation also initiates from the gas pore, and a cluster of gas pores is observed along the crack propagation direction. In contrast, the BM generally exhibits a single surface crack origin without obvious defects under low-stress conditions. Moreover, the fatigue crack growth region generally occupies a larger fraction of the fracture surface in the BM than in the welded joints, which is consistent with the longer fatigue life of the BM. These observations indicate that gas pores introduced during welding play a dominant role in crack initiation and make an important contribution to the reduced fatigue life of the welded joints [18], i.e., gas pores are an important factor responsible for the poor fatigue performance of the Ti-6Al-4V ELI titanium alloy welded joints.

### 3.3. Fatigue Crack Growth Behavior

#### 3.3.1. Pure Mode I Fatigue Crack Growth

**Figure 7:**
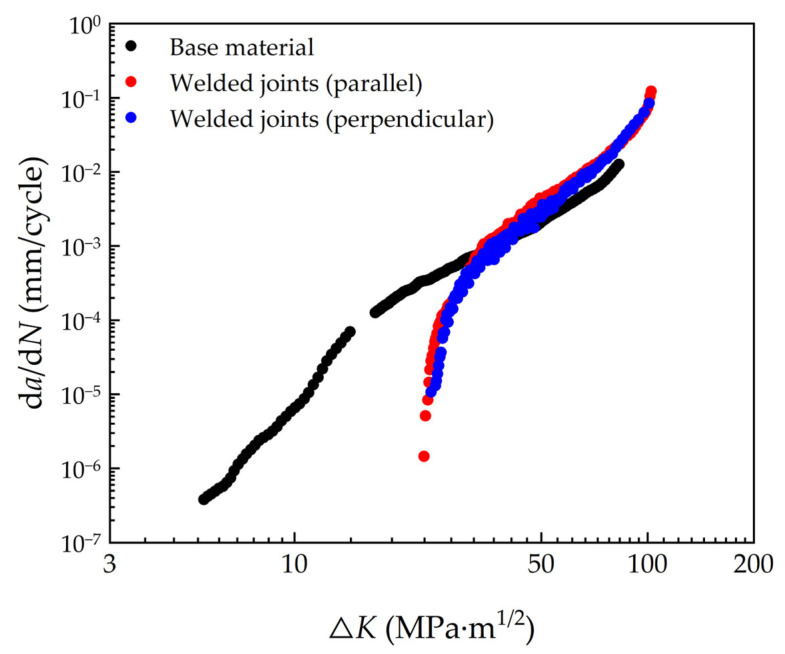
Comparison of da/dN−ΔK data of the BM and the welded joints with crack planes parallel and perpendicular to the welding direction under pure mode I loading.

Figure 7 compares da/dN−ΔK data of the BM and the welded joints under pure mode I loading. For the welded joints, two different crack orientations relative to welding direction were considered: one in which the crack plane was parallel to the welding direction and one in which it was perpendicular to the welding direction. In the parallel configuration, the crack tip remained within the FZ throughout crack growth, whereas in the perpendicular configuration, the crack sequentially traversed the FZ and the HAZ on both sides.

It is seen that the fatigue crack growth rate with stress intensify factor range are very similar for the two different crack orientations relative to welding direction, indicating that the orientation of the crack plane relative to the welding direction has a negligible effect on the overall crack growth rate. Compared with the BM, the welded joints require a higher ΔK in the low crack-growth-rate region, but the two sets of data gradually approach each other as da/dN increases and becomes nearly coincident at about $10^{-3}$ mm/cycle. This higher near-threshold resistance may be associated with the acicular $\alpha^{\prime }$ martensitic structure in the FZ. Lamellar or colony-type microstructures increase crack-path tortuosity and enhance crack growth resistance, particularly in the near-threshold region [19,20,21]. At higher ΔK, the macroscopic mechanical driving force becomes dominant, and the influence of the microstructural differences is correspondingly reduced so that the crack growth data of the welded joints and the BM tend to converge. Therefore, the welded joints show relatively higher crack growth resistance than the BM in the near-threshold region under pure mode I loading.

#### 3.3.2. Mixed-Mode I–II Fatigue Crack Growth

**Figure 8:**
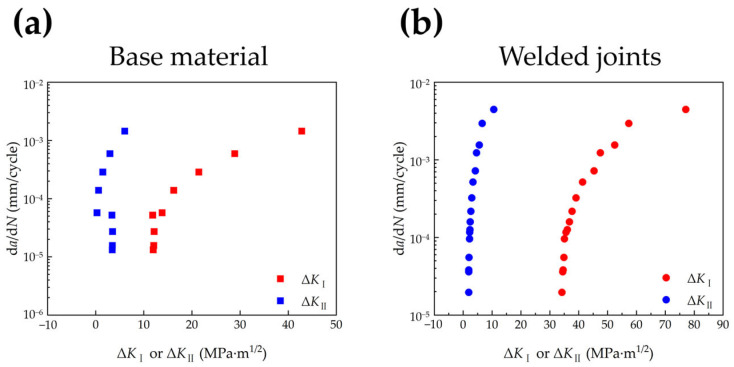
Relationships between da/dN and stress intensity factor range components $\Delta K_{I}$ and $\Delta K_{II}$ for the BM and the welded joints under mixed-mode I–II loading: (**a**) BM; (**b**) Welded joints.

Figure 8 shows the relationships between the crack growth rate da/dN and the stress intensity factor range components $\Delta K_{I}$ and $\Delta K_{II}$ for the BM and the welded joints under mixed-mode I–II loading. It is seen that the BM exhibits lower $\Delta K_{I}$ values at the same crack growth rate. For example, the corresponding $\Delta K_{I}$ values for the BM is approximately 16.2 MPa·$m^{1/2}$ at $da/dN\approx 1.0\times 10^{-4}$ mm/cycle. While the welded joints require about 2.2 times higher $\Delta K_{I}$ than the BM at this crack growth rate.

To compare the crack growth behavior under pure mode I and mixed-mode I–II loadings, the equivalent stress intensity factor range is conventionally used to characterize the mixed-mode crack driving force [22,23]:

(3) $$\Delta K_{eq}=\sqrt{{\Delta K_{I}}^{2}+{\Delta K_{II}}^{2}}$$

**Figure 9:**
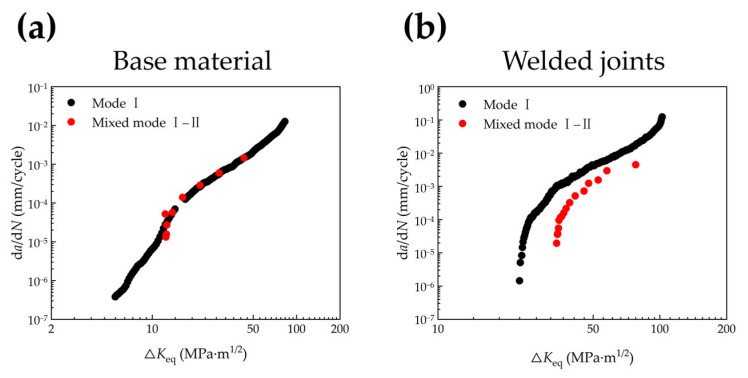
Comparison of $da/dN-\Delta K_{eq}$ relationships between CT and CTS data: (**a**) BM; (**b**) welded joints.

The corresponding results are shown in Figure 9. For the BM, the mixed-mode data agree well with the pure mode I crack growth data, indicating that the conventional equivalent stress intensity factor range is suitable for describing the crack growth behavior in BM under mixed-mode I–II loading. For the welded joints, however, the mixed-mode data lie entirely to the right of the pure mode I data and cannot be correlated using the same definition of $\Delta K_{eq}$. This result indicates that the conventional equivalent stress intensity factor range is insufficient to characterize the crack growth driving force of the welded joints under mixed-mode I–II loading.

To account for this effect, the conventional equivalent stress intensity factor range was modified by introducing attenuation coefficient ϕ that depends only on the mode mixity ratio defined by $\Delta K_{II}/\Delta K_{I}$ in this paper:

(4) $$\phi =\frac{1}{1+{\left(\frac{\Delta K_{II}}{\Delta K_{I}}\right)}^{p}}$$

where p>0 is a dimensionless parameter. The coefficient ϕ ranges from 0 to 1 and approaches 1 as $\Delta K_{II}\to 0$.

The modified equivalent stress intensity factor range is then written as

(5) $$\Delta K_{eq}=\sqrt{\phi \cdot {\Delta K_{I}}^{2}+{\Delta K_{II}}^{2}}=\sqrt{\frac{{\Delta K_{I}}^{2}}{1+{\left(\frac{\Delta K_{II}}{\Delta K_{I}}\right)}^{p}}+{\Delta K_{II}}^{2}}$$

**Figure 10:**
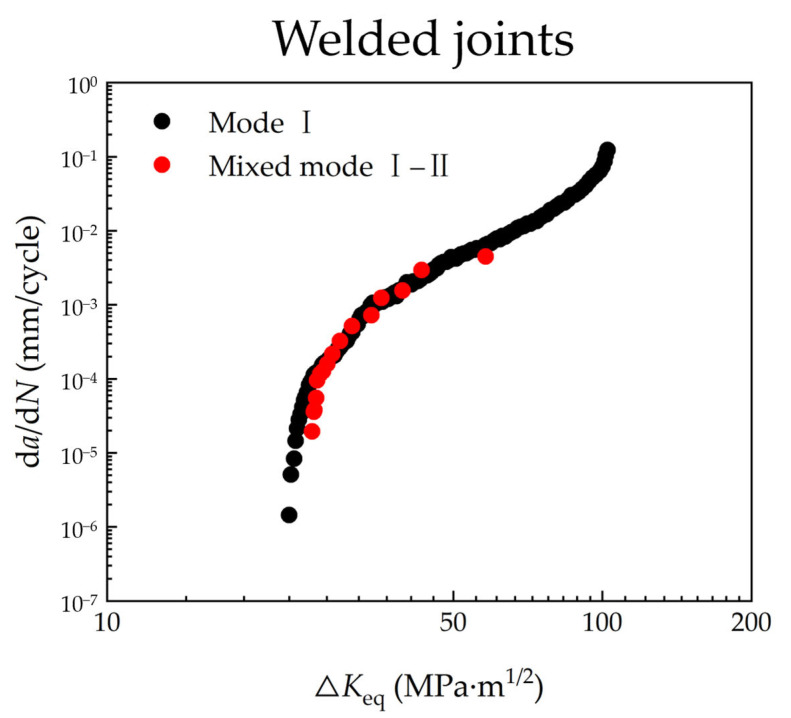
Correlation of the pure mode I and mixed-mode I–II crack growth data of the welded joints in the $da/dN-\Delta K_{eq}$ form using the modified equivalent stress intensity factor range model.

Under pure mode I loading, the modified equivalent stress intensity factor range reduces to $\Delta K_{I}$, which is consistent with the common definition for pure mode I crack. The parameter *p* was determined by fitting the mixed-mode I–II crack growth data to the pure mode I data in the da/dN − $\Delta K_{eq}$ form. The value p = 0.1 gave the closest correlation between the corrected mixed-mode data and the pure mode I data, and was therefore adopted in this study. Figure 10 shows the correlation between pure mode I and mixed-mode I–II crack growth data of the welded joints in the da/dN − $\Delta K_{eq}$ relation obtained using the modified equivalent stress intensity factor range model. The results show that the modified model improves the correlation between the two sets of data well.

## 4. Conclusions

The axial fatigue behavior, pure mode I fatigue crack growth, and mixed-mode I–II fatigue crack growth of Ti-6Al-4V ELI titanium alloy welded joints were investigated and compared with those of the BM. The main conclusions are as follows:

1. The welded joints exhibit lower fatigue strength than the BM, but with a similar slope for the *S–N* curve. SEM observation shows that the fracture mode depends on the applied stress level: specimens under high-stress show ductile fracture features without crack initiation and growth region, whereas specimens under intermediate and low-stress mainly initiate from gas pores or pore clusters.
2. Under pure mode I loading, the welded joints require a higher ΔK than the base material in the low crack-growth-rate region, and the difference decreases while increasing the crack growth rate. When the crack growth rate reaches approximately $10^{-3}$ mm/cycle, it becomes comparable between the welded joints and the BM.
3. Under mixed-mode I–II loading, the welded joints require higher stress intensity factor range components $\Delta K_{I}$ than the BM. The conventional equivalent stress intensity factor range is insufficient to correlate the pure mode I and mixed-mode I–II crack growth data of the welded joints. By introducing a parameter dependent on the mode–mixity ratio into the expression of equivalent stress intensity factor range, the modified model correlates the crack growth data well under the two loading conditions.

The findings indicate that welding-induced gas pores mainly affect the fatigue performance of the welded joints through crack initiation, while the welded joints still show relatively high crack-growth resistance in the near-threshold region. These results provide help for the fatigue design and damage-tolerant assessment of Ti-6Al-4V ELI titanium alloy welded structures.

## Figures and Tables

**Table 1:** Chemical composition of Ti-6Al-4V ELI titanium alloy.

Elements	Ti	Al	V	Fe	C	H	O	N
wt.%	Bal.	6.47	4.22	0.20	0.0046	0.0022	0.12	<0.003

**Table 2:** Mechanical properties used in finite element analysis.

Material	Elastic Modulus/GPa	Poisson’s Ratio
Ti-6Al-4V ELI BM	122.1	0.34
High-strength steel	210	0.30

## Data Availability

The data presented in this study are available on request from the corresponding author.
